# The Role of Autonomy in Multi-Partnered Relationships

**DOI:** 10.1007/s10508-025-03202-6

**Published:** 2025-08-01

**Authors:** Sonja Bröning

**Affiliations:** Faculty of Human Sciences, Medical School Hamburg, University of Applied Sciences and Medical University, Am Kaiserkai 1, 20457 Hamburg, Germany

**Keywords:** Polyamory, Consensual non-monogamy, Autonomy, Qualitative interviews, Reflexive thematic analysis

## Abstract

Polyamory is a form of consensual non-monogamy (CNM) that openly endorses multiple romantic and sexual relationships. Previous research has reported high satisfaction and need fulfillment in polyamorous relationships. However, the feasibility of polyamory for a wider population given the societal monogamy norm is highly debated. Desire for relational autonomy, such as the freedom to love multiple partners and be sexually intimate with them, is a widespread motive for engaging in CNM. In this qualitative study, we explored if and how this kind of autonomy was experienced by polyamorous individuals in daily life. Autonomy in relationships was conceptualized through the differential lenses of self-determination theory, attachment theory, and differentiation theory. We conducted 20 semi-structured interviews with German-speaking polyamorists. The material was analyzed using reflexive thematic analysis to answer the following research questions: (1) In what ways is personal autonomy created and expressed in polyamorous relationships? (2) Are autonomy-related needs fulfilled in polyamorous relationships? (3) How important is personal autonomy for polyamorous individuals? Overall, participants reported a high need for and level of “doing autonomy” in their polyamorous relationships. Four central aspects of this autonomy emerged: assertive communication, independent emotion regulation, self-congruent lifestyle, and valuing freedom. Different nuances of these four aspects are discussed with regard to the study’s research questions and to their implications for mental health professionals working with polyamorous clients.

## Introduction

Polyamory is a form of consensual non-monogamy (CNM) that welcomes multiple romantic and sexual relationships. In a society where monogamy is viewed as the norm, CNM can be viewed as an expression of relational diversity. CNM relationships share the commonality that all partners involved explicitly agree on the option of having more than just one partner. Multi-partnered relationships can vary on many factors, including the kinds of relationships engaged in (romantic or only sexual, long term or short -term), the degree of transparency and disclosure involved, and the specific terms of consensually agreed behavioral conduct between partners (Mogilski et al., 2023). For the USA and Canada, the general prevalence for CNM is estimated to be around 4–5% (Conley et al., 2013a, 2013b; Fairbrother et al., 2019; Moors et al., 2015). CNM is more common in persons from sexual minorities, with percentages ranging from 32 to 45% in individuals identifying as “gay,” from 5 to 35% in individuals identifying as “lesbian,” and from 22 to 48% in individuals identifying as “bisexual” (Cubells-Serra et al., 2021; Haupert et al., 2017; Levine et al., 2018). Few data exist for Germany. In a recent survey, lifetime prevalence for an open relationship was 14% for males and 7% for females, while under age 40, it was 19% of male and 10% of female respondents (Elitepartner, 2023). CNM has been discussed as an option for escaping the pressure of a lifelong monogamy norm that places high expectations around need fulfillment from one partner. Unmet sexual, emotional, or social needs cause relational breakups that are followed by a wake of severe consequences for adults and children affected by them. Potential risks such as instability and fragility of CNM have also been described, especially given limited structural, financial, and time-related resources (Kauppi, 2021). Since monogamy is the cultural norm in western societies, individuals who do not conceal their CNM relationships are frequently stigmatized as “amoral” or deficient (Thompson et al., 2018).

Polyamory is often viewed as the most extensive CNM model, explicitly allowing not only erotic encounters, but also romantic and relationship components. Polyamorists challenge monogamous norms by stating that theirs is an “ethical” way of conducting more than one love relationship, compared to cheating and infidelity. Values such as commitment, honesty, consensus, open communication, and trust play an important role in polyamory discourses (Katz & Katz, 2022). Multi-partner constellations exhibit high levels of relationship satisfaction (Garner et al., 2019), sometimes higher than those of monogamous individuals (Conley et al., 2013a, 2013b), and high satisfaction with need fulfillment (Mitchell et al., 2013). However, due to a lack of longitudinal data, we know little about the long-term relationship satisfaction in polyamorists. Since intimacy and closeness with simultaneous partners must be maintained in the face of non-exclusivity, negative affect such as jealousy or dissatisfaction could prevail in the long run (Wetzel, 2014), for instance in persons with attachment insecurities. To date, it remains unclear who benefits in multi-partnered constellations, and who does not.

One differentiating factor may be the need for autonomy. Polyamorous people are not required to maintain sexual exclusivity with one partner. Thus, their autonomy appears exceedingly high in terms of freedom to shape their personal love lives compared to monogamous relationships. This may be appealing to persons with a high need for autonomy, motivating them to enter into such a form of relationship (Hnatkovičová & Bianchi, 2022). On the other hand, polyamory often presents as a highly negotiated relationship model, fraught with terms and conditions that limit personal freedom (Kauppi, 2021). Beyond an individualistic point of view, multiple academic viewpoints, e.g., feministic, philosophical sociological, and queer perspectives, have situated the question of relational autonomy in the larger context of societal (class, gender, race) differences and their interaction (see Klesse, 2018, for a detailed overview). Feminists have viewed CNM as a pathway toward women’s erotic agency through the rejection of the (productive, emotional and sexual) inequalities experienced by women within the institution of marriage, or toward sexual and gender-related freedom for queer minorities (e.g., Deri, 2015; Klesse, 2005; Schippers, 2016). Other positions have argued that erotic autonomy is an illusion as long as hetero-/mono-normative structures organize intimacy and permeate emotions in ways that reinforce patriarchal oppression of non-white, non-male heterosexual identities, frequently intersecting with each other (e.g., Veltman & Piper, 2014). From a philosophical perspective, Gregoratto (2020, 2021) argued that while polyamory per se is not free of coercion and oppression, experiencing “friction, tension and conflict between the (alleged) ethical order and the affects that resist assimilation” (p. 257) in polyamorous relationships (such as compersion instead of jealousy) may precisely be the place where transformation toward subjective freedom originates. Here, I use qualitative data to explore the subjective experience of personal autonomy in daily polyamorous life from a psychological perspective on relational autonomy, using the lenses of self-determination theory, attachment theory, and differentiation theory.

### Autonomy in Relationships Viewed Through the Lens of Self-Determination Theory

Self-determination theory (SDT) places autonomy in the context of three basic needs, that form each and every individual’s (self-)motivation and well-being: autonomy, relatedness, and competence (Ryan & Deci, 2000). The extent to which these needs are met relates directly to an individual’s sense of well-being. The need for competence is satisfied by the feeling of being able to successfully produce desired effects and outcomes in various domains. Relatedness results from the experience of feeling close and connected to significant others. Subjective autonomy involves the perception that one’s activities and environment are matched and endorsed with the self. In other words, personal autonomy exists when actions and life situations are experienced as “self-governed”: self-initiated, self-directed, and consistent with personal values and needs (Ryan & Deci, 2000; Ryan et al., 1997). Thus, personal autonomy is reduced and threatened by coercion, threat, forced behavior, and external control.

Autonomy (or the absence of autonomy) can be experienced in intimate relationships where the shifting, dialectic tension resulting from the bipolarity of autonomy and connectedness must be balanced through “simultaneous differentiation-from, yet fusion-with, the Other” (Baxter, 2006, p. 101). Evolving life situations and individual needs constantly challenge this delicate balance, often leading to breakup. Some view this as inevitable in a highly individualized society where self-development and romantic connection can appear as equally desirable, but often contradictory goals (Reckwitz, 2019).

The effective handling of disagreement and conflict within close relationships requires assertive reasoning while simultaneously preserving the closeness of the relationship in communication (McIsaac et al., 2008). Empirical work examining interaction styles showed that behaviors promoting autonomy (i.e., using reasoning and expressing confidence) and relatedness (i.e., showing warmth, collaborative behavior) are not opposed, but positively correlated in close relationships (Allen et al., 1994, 2000; Taradash et al., 2001). Individuals who operate under a relationship style of equality characterized by both high autonomy and high connectedness have reported high levels of mental health and satisfaction compared to persons low on autonomy in relationships, who are more likely to be dissatisfied (Neff & Harter, 2003).

In sum, SDT explains why a subjective sense of autonomy is important: because of the satisfaction resulting from feelings of agency when individuals’ actions match their personal needs and values. SDT highlights the importance of autonomy as self-advocacy in relational interactions, and how this can and must be balanced with relatedness.

Some research points to autonomy in CNM relationship interactions. A culture of assertive, open communication has been reported (Mogilski et al., 2023; Wosick-Correa, 2010). Needs and relationship realities as well as relationship rules and agreements are viewed as negotiable among all partners at eye level. Polyamorists strive to achieve full consent from everyone concerned (Barker, 2005; Klesse, 2007). Andersson (2022) points out the amount of moral work her interviewees invest into reaching consensuality in contrast to the secrecy involved in infidelity. Recent publications on clinical work with polyamorous constellations stress the fact that a high degree of social intelligence is necessary to navigate the complex autonomy-connection balance in multi-partnered relationships (Kauppi, 2021; Vaughan & Burnes, 2022).

### Autonomy in Relationships Viewed Through the Lens of Attachment Theory

Individuals differ in their need for autonomy and their preferences around closeness in intimate relationships. Attachment theory places these differences in a developmental context. The innate, universal attachment system motivates infants from birth to seek the proximity of their caregivers when they feel threatened or uncomfortable (Bowlby, 1979). When caregivers respond consistently, negative affect is regulated (Ainsworth et al., 2015). Countless everyday interactions between caregiver and child shape typical patterns of thought, emotion, and behavior in close relationships. These are rooted in physiology and affect, experienced, for instance, in an elevated stress sensitivity when a person’s early attachment experiences were not reassuring, or in a less rewarding sense of relief through social reassurance in threatening situations (Feldman, 2017; Luyten et al., 2020). In this way, the capability for self-soothing and co-regulation with significant others is influenced by a person’s attachment security.

Romantic relationships extend childhood attachment relationships into adult life (Fisher, 2000; Hazan & Shaver, 1987). Over time, a romantic partner becomes an increasingly important source of psychological security, similar to the caregiver in a parent–child-relationship (Treboux et al., 2004). Needs for closeness and autonomy in intimate relationships are influenced by the attachment orientation of each dyad member, and the dynamic they unfold in their interaction. A person with a secure attachment orientation generally feels emotionally close to a romantic partner, believes that this closeness will be reciprocated, and perceives neither proximity nor autonomous behavior as threatening. Insecure attachment in adults is described along the dimensions of attachment anxiety or avoidance. Attachment anxiety is characterized by fear of rejection, abandonment and being left alone, and by a tendency to frequently call on significant others for emotional support, while doubting their availability. Attachment-related avoidance is characterized by discomfort with closeness and dependence, a tendency to withdraw and self-regulate under stress (and also to expect significant others to cope in this way), and to generally strive for high levels of personal autonomy (Mikulincer & Shaver, 2012). Importantly, autonomy in attachment theory is not viewed as something persons with attachment avoidance have the most of. Instead, autonomy is mostly conceptualized as emotional autonomy, i.e., being comfortable with feeling both closeness and independence. Emotional autonomy is a key ingredient of attachment security, which is therefore classified as “secure-autonomous” in the Adult Attachment Interview (Main et al., 2008).

Attachment insecurity affects a wide range of relationship outcomes, such as relationship satisfaction and dyadic coping (Bröning & Wartberg, 2022; Gagliardi, 2021; Givertz et al., 2013). It also influences sexual behavior (Birnbaum & Reis, 2019). Studies have reported that attachment insecurity is associated with lower sexual satisfaction, less consensual sex, negative body self-concept, and more sexual problems. Attachment anxiety is correlated with erotophilia, and especially with the sex motives “closeness,” and “wish for physical proximity,” as well as more feelings of shame, guilt and anxiety in partnered sexuality. Attachment avoidance is connected to having more casual sex and can be linked to sex-motives that are not romantically motivated such as manipulation of a partner or status motives (Stefanou & McCabe, 2012). It has also been linked to infidelity (DeWall et al., 2011).

In sum, attachment theory explains differences in the individual need for closeness and distance in romantic relationships based on developmental experience. An autonomous individual is emotionally comfortable with both dependence and independence and is able to use both self and other for psychological comfort and security.

Even though conceptualizing romantic love as extension of the mother–child bond has sometimes reinforced the ideal of monogamy, attachment theory and polyamory do not contradict each other. Attachment theory is dyadic, but it encompasses the ability to have close simultaneous bonds with several persons (i.e., mother and father). However, polyamory may require a high degree of self-regulation and security. For instance, being in a polyamorous relationship can mean spending substantial amount of time alone, watching a partner leave for a romantic weekend with someone else, or being exposed to “new relationship energy” of partners, when they fall in love with a new acquaintance. This type of experience can be accompanied by intense negative affect such as jealousy, envy, and fear of loss. According to Fern (2020), individuals with high attachment anxiety might be affected by feelings of mistrust up to panic in polyamorous relationships, where conflict and breakup can appear imminent. Consequently, researchers have pointed out the importance of attachment security in CNM (Katz & Katz, 2022). Existing research has found that individuals in CNM relationships generally show high degrees of attachment security, indicating that polyamorous individuals feel comfortable with independence and intimacy (Bricker & Horne, 2007; Moors et al., 2015; Morrison et al., 2013). However, research is inconclusive: in one study, highly avoidant individuals showed positive attitudes toward and willingness to engage in CNM (Moors et al., 2015), but were less likely to do so. In another study, individuals with attachment anxiety (identifying as lesbian, gay, bisexual, or pansexual) held negative attitudes about CNM, yet were more likely to be in a CNM relationship (Moors et al., 2017).

### Autonomy in Relationships Viewed Through the Lens of Differentiation Theory

Differentiation theory adds another nuance to the concept of autonomy. It developed independently from attachment theory and points to the importance of a self-validated (i.e., autonomous) sense of self (Hardy & Fisher, 2018). “Differentiation” is a developmental process by which individuals learn to balance the human drives for both deep connection and unique identity (Kerr & Bowen, 1988). Unique identity can be experienced when “someone makes more deeply or truly hers those dimensions of her biography that constitute or manifest her agency or her identity” (Friedman, 1998, p. 169). Similar to SDT and attachment theory, this line of theory views autonomy and its expression as deeply social in character. Kernis and Goldman (2006) argue that autonomy represents an essential component of authenticity, as one is “acting in ways congruent with one’s values, preferences, and needs,” whereas inauthenticity entails “acting merely to please others or to attain rewards or avoid punishments” (p. 302). Thus, congruent self-endorsement of behavior is central to the definition of autonomy, as well as representing a core characteristic of authenticity (Ryan & Ryan, 2019). A person’s unique identity can be compromised or threatened within a relationship, for instance through the predomination of a partner’s interests, needs, decisions, or long-range plans (Friedman, 1998).

The ability to “hold your own” in relational situations of tension is seen as the direct result of a sufficiently high level of differentiation. It includes the capability to reflect on own character and how it can be represented in the surrounding society, and to self-validate when this representation is challenged. According to Schnarch (2009), persons low on differentiation are “high in fusion” in intimate relationships. They depend on their partners for acceptance and validation. Attachment-based approaches emphasize emotional connection and couple co-regulation as means to improve romantic relationships. Differentiation-based approaches emphasize the acceptance of difference. They also view the ability to self-disclose and self-regulate as key ingredients to successful long-term love, because this broadens the dyads capacity for balancing autonomy and connection (Schnarch, 2009). Some studies have found associations between high levels of differentiation and mental health, sexual and relationship satisfaction (Hardy & Fisher, 2018; Lampis, 2016).

In sum, differentiation theory views autonomy as having a sense of one’s own “unique identity” and living by this identity while at the same time accepting other persons uniqueness and difference. A person’s ability to act in congruence with their own identity is strongly influenced by their ability to self-validate and self-regulate when this lifestyle is challenged.

Polyamory can be viewed as a learning field for differentiation, since total fusion with only one person is inherently impossible in a multi-partnered situation. At the same time, stigma from the outside, i.e., the monogamous society, is a constant identity threat within CNM (Balzarini et al., 2018; Schechinger et al., 2018). Belonging to a minority has been conceptualized as stressful through multiple incidents of victimization and discrimination (Meyer, 1995, 2003). Related to this, psychological vulnerabilities have been consistently found in persons with sexual and gender diversity (Flentje et al., 2020; Plöderl & Tremblay, 2015; Reynish et al., 2023). Pieper and Bauer (2014) discuss polyamory as ethical model that helps autonomous individuals resist the mono-normative matrix and undermine existing power structures threatening unique shapes of identity. Sheff (2020) emphasizes the special potential of CNM for women, for whom engaging in parallel sexual relationships for the purpose of personal pleasure and satisfaction is particularly sanctioned in the traditional monogamous role model. Thus, polyamory may provide a framework for differentiation in the face of identity challenges.

### Research Questions

The need for autonomy appears to be a powerful motivation for entering into a polyamorous relationship. Hnatkovičová and Bianchi (2022) identified eight motives for polyamory: “personal growth and autonomy,” “fulfillment of needs not met in a monogamous relationship,” “identity development in polyamory,” “expression of political values,” “exploring minority identities (sexual fluidity and bisexuality),” “desire for sexual diversity,” “need to belong to a community,” and “psychodynamic reasons.” The first six motives fall more or less squarely into the field of autonomy, comprising the self-directed fulfillment of personal needs, exploring one’s own identity, and living in congruence with personal values and goals. But how does this vision of personal freedom and boundless love deliver in daily life? Does polyamory provide an arena where autonomy is experienced in everyday life, given the societal norms and restrictions mentioned above? This work explores autonomy in multi-partnered relationships along the following research questions:

1. In what ways is personal autonomy created and expressed in polyamorous relationships?
2. Are autonomy-related needs fulfilled in polyamorous relationships?
3. How important is personal autonomy for polyamorous individuals?

## Method

### Participants

The empirical material for this analysis is an interview study on polyamory conducted in 2021 and 2022 with the help of two psychology master students, one of whom (Klara Gebhard) is co-authoring a publication elsewhere based on her master’s thesis (Bröning et al., 2024). However, the present analysis is my own, so I will refer to the study as mine for the purposes of this article.

In total, we interviewed 20 individuals who were explicitly involved in a polyamorous relationship. The interviewees were recruited by a call for participants living a polyamorous lifestyle posted by the researchers on Instagram and then snowballed to other community networks, social media platforms, and chat groups. Eligibility for inclusion was defined as the existence of at least two concurrent romantic relationships conducted in consensus with all intimate partners. This excluded other forms of CNM, such as “swinging,” “don’t ask don’t tell = DADT,” or open relationships restricted to sexual but not romantic encounters outside a romantically exclusive relationship. All participants provided written consent for the study and audiotaping interviews for transcription.

We recruited a relatively young sample, with interviewees’ age ranging from 20 to 48 years (*M* = 33.15 years, SD = 8.87). The majority of interviewees identified their gender as female (*n* = 13), followed by male (*n* = 4), non-binary (*n* = 2), and genderqueer (*n* = 1). Eight respondents label their sexual orientation as bisexual, with two of these additionally identified as heteroromantic. The remaining participants describe themselves as pansexual (*n* = 7), heterosexual (*n* = 2), although one participant reports being unsure of this, as well as queer (*n* = 1), fluid (*n* = 1), and heteroflexible (*n* = 1). Five participants were married, six had children. Half of the respondents (50%, n = 10) were living with a nesting partner at the time of the survey. This nesting partner corresponded with “partner 1” (being the most prominent attachment figure) in all cases. “Partner 1” also was parent to the children in all cases. Thus, even though relationship duration was not explicitly inquired about (which is a limitation of this study), we can assume from the data that the relationship with “partner 1” usually was the longer-standing connection.

### Measures and Procedure

The interviews were conducted online via videoconferencing, mostly due to COVID restrictions, but also because participants came from different regions in Germany. We asked our participants to find a quiet, private setting for the interview. The interview language was German, and the interviews lasted for 60–90 min. The interviews were recorded and transcribed verbatim; interjections like “uhm,” “yes,” “well,” and so on were included, but we did do a slight smoothing of broad dialects to make the text more readable. The interview sections used here were translated by myself. We used a semi-structured approach with open-ended questions aimed at capturing polyamorous experiences regarding (1) motivation for polyamorous lifestyle, (2) attachment-related experiences of intimacy, emotional closeness, nurturance and support, (3) questions of distance, autonomy, rules, and boundaries, and (4) advantages and disadvantages of polyamorous lifestyle. Since our main interest at the time was to capture differences in attachment-related interactions between an individual and their simultaneous intimate partners, a comparative approach was adopted in the interview design (see also Balzarini et al., 2019). We asked our interviewees to define their closest attachment figure (operationalized as the person who would be contacted first in the event of a personal crisis), who was then labeled “partner 1.” We then asked our interviewees to define the second most important intimate relationship partner, who was labeled “partner 2.” We asked questions b) and c) for both partners (e.g., how do you feel about nurturance and support in relationship with partner 1? And with partner 2?), thus contrasting these two relationships regardless of the possible existence of further relationships. Here, I use fictive names for readability and to protect our interviewees’ identities.

### Data Analysis

To answer my research questions regarding the role of autonomy in polyamorous relationships, which arose from an earlier analysis of the data (Bröning et al., 2024), I chose an inductive approach of coding and theme development directed by the content of the data. This approach reflects a social constructionist stance that explores how social processes and interactions (Hacking, 1999) as well as the subjective meaning ascribed to them shape and construct reality. All interviews were tape-recorded and transcribed. To analyze the interview transcripts, I used reflexive thematic analysis following Braun and Clarke (2006). I read and re-read the material guided by the psychological qualities of autonomy described in the theories above, especially expressions of agency, of self-advocacy in interactions (SDT), emotional ease at handling both closeness/ dependence and distance/ independence (attachment theory), a sense of congruence with one’s unique identity, which is self-validated and can be sustained through self-regulation when challenged (differentiation theory; Step 1). I systematically coded passages of interest (Step 2) and collated them into potential themes. These included “addressing and discussing difficult topics,” “standing up for myself,” “authenticity,” “exploration,” “self-actualization,” “freedom,” "boundaries,” “differentiation,” “responsibility for emotions,” and “explicitly valuing autonomy.” To complement this initial theoretical approach, I searched for all instances in the material where autonomy and (in)dependence are mentioned (explicitly or implicitly), to see how this is brought up by the interviewees. This more inductive approach contributed additional perspectives on how and in what situations the interviewees brought up and reflected on the value of autonomy (Steps 3 and 4). Combining these approaches, re-reading the material, and reexamining the themes, I grouped the relevant material under four distinct themes around autonomy in polyamorous relationships (Steps 5 and 6): (1) Assertive communication, (2) Independent emotion regulation, (3) Self-congruent lifestyle, (4) Valuing Freedom.

#### Positionality

The interpretation of these themes was conducted by a process of reading and re-reading, as well as reference to relevant literature and consultation with colleagues. No claims are made for the generality or representativeness of the sample. Throughout this process, my positionality as a white, married, middle-aged, middle-class mother of two children must be acknowledged. It inevitably shapes my perspectives, biases, and interaction with the material at every research stage. Coming from a binational family, and having worked in economics prior to my current career path, I appreciate multiple perspectives and the complexities inherent in socialization. My identity as a woman may impact sensitivities toward gender dynamics and power structures within relationships. My academic journey in educational science, sociology and psychology has equipped me with a critical lens through which I view social phenomena. Professionally, I have consistently combined research with clinical work in community-based prevention projects and in my own private relationship therapy practice. This experience has heightened my awareness of the socioeconomic and political factors that affect marginalized communities. I strive to remain reflexive, interrogating how my biases and assumptions may shape the research, e.g., through consultation with colleagues and the target group of my research. In this case, for instance, I presented the identified themes to a group of polyamorous individuals with whom I was conducting workshops, which rendered further aspects of interpretation.

## Results

### Assertive Communication

A first theme that came up frequently in our participants’ accounts of their polyamorous lifestyle was an assertive communication style. Autonomy was expressed and balanced in several different ways. For one thing, participants repeatedly mentioned announcing their own intentions and relationship needs in a self-confident manner. Christine (she/her, 36, bisexual) shares that she had made an announcement from the very start:

Christine: I told him from the beginning “Hey, I think I'm not a monogamous person, except maybe for a while now for both of us.” Because, somehow, I think it was also a very deeply felt affection and love for this person, out of which I felt, oh yes, I want to give my resources to this right now. But it was somehow clear that the signs were pointing to something else for me. And then, sometime after one and a half or two years, I said if it would be possible to open up this relationship. There was a specific reason for that then, but it was also announced from the very beginning.

It is interesting how this is expressed. Instead of saying “after two years, I asked if would be possible,” she phrases it as “I said if it would be possible,” thus demonstrating assertiveness by placing it somewhere between a unilateral announcement and a question. Nicole (she/her, 39, pansexual) also “said right from the start that I would like to try this [polyamorous relationship model] out to see if it fits.” Lisanne (she/her, 20, bisexual) reports announcing to her partner that she would like to gain more experience with women, to which he replied “well, if you want to experience that, go for it.” Barbara (22, she/her, bisexual/heteroromantic) mentions that there is a lot going on in her life with traveling and spending time with two partners. Occasionally, she will announce to a demanding partner: “Oh no, I'm going to drive home in the evening and I'm not sleeping here now. And I kind of really need a day or two for myself.”

Second, participants appear very proactive in initiating discussion about difficult topics. They have an open, resolution-oriented conflict style and are not shy or hesitant in bringing up problems in a direct, confrontative and timely manner. Pia (they/them, 42, pansexual) shares:

Pia: I'm the one who thinks of more problems that can arise, and anticipates them. Exactly, and I just talk about it and then they say "Yes, mhm (affirmative)" and then they think about it and then they usually say "Yes, I agree" or "Yes, it's good that you mention it, maybe we can do something like this." So then it is addressed and a compromise is found and then it works.

Thirdly, not only do participants initiate discussion, they also promote autonomy in their conversation partner by wanting to hear the other side, by asking questions about how a partner perceives the issue at hand. Michelle (she/her, 41, bisexual) reports that being inquisitive is an important way in dealing with insecurity about closeness:

Michelle: When […] I hear something from another woman and I stumble upon it, I just ask "does that make a difference for us? At what point does it make a difference? What is important to you there? (...) What feels weird to me right now?"–like that.

Also, when her wife is jealous, she reports feeling curious and wanting to know more:

Michelle: So then, I ask what she thinks where the jealousy comes from. If it's something specific or not, if it's because it's a man, if it's because of a certain [sexual] technique, if it's because of this person or what exactly my contribution is toward making her feel jealous.

Finally, to maintain connection with their significant other in light of assertiveness, participants report that they engage in frequent disclosures of emotions. Communication of emotions, the exact meaning of emotions such as sadness, envy, or jealousy, and the vulnerability caused by emotions contain the reason for the need or intention at hand. Christine describes feeling jealousy and responds to the question about how she deals with it:

Christine: I usually just say it then. (...) Jealousy is often a bit of fear or sadness for me. I think, I try to communicate what exactly it is at that point. [ …] And that way it doesn't build up so much. I think there is little fear of losing the respect or affection of the other person, as I notice. This fear is very, very, very small.

Alexis (they/them, 30, pansexual) and their partner developed a color code for the disclosure of emotions and communication about them:

Alexis: So I would say, between jealousy and compersion, it's a spectrum. (laughs). And with regard to my roommate, we've sort of introduced a system to indicate how okay a situation is for me right now. So, for example, if I'm really happy when they're cuddling or something, then I just say "green." (laughs) And (...) for example (...) if [partner 1] makes a joke that goes in a sexual direction, then I can say "dark gray," that's on the border. And if something is already over the border, then just “black.”

A fifth aspect of autonomous behavior in interaction is “working it out,” i.e., persistently engaging in communication in a tenacious, conscious effort to work through discord. This kind of communication was termed “deep talk” by one participant. Many polyamorous situations started with intense and prolonged discussions about opening the relationship. Prolonged discussion is also part of daily life in resolving conflict. Kim (they/them, 45) states: “I resolve conflict by trying to talk until both partners feel seen and we both know what the issue is and then a solution can be found that is acceptable to both.”

### Independent Emotion Regulation

Autonomous emotion regulation was another common theme described by the participants of our study. The first observation are behaviors of non-reactivity and self-soothing. Non-reactivity, a term from Acceptance and Commitment Therapy (Hayes et al., 1999), refers to accepting an experience without immediately acting upon it, which encourages individuals to fully experience their bodily sensations, emotions, and thoughts without changing or avoiding them. In our data, participants frequently describe that they wait and figure out their experience before reacting in the immediate situation, like Lukas (he/him, 28, pansexual):

Lukas: I need a lot of time to think of the words I actually want to use, because I always have a lot of trouble finding the right words for the situation. But by now we have balanced this in such a way that when there is a fight and I start feeling overwhelmed in the situation, we leave the conversation for the time being, I go to my room, because we have separate rooms, and think about "Okay, what do I actually want to say now? How do I feel about this topic?" and then I approach her again and we talk about it calmly.

Lisanne also shares that she does not spend a lot of time with her second partner, so she tries a bit harder to watch herself and stay calm so as not to ruin the few available hours with him. Participants describe a variety of self-soothing strategies in stressful situations with intimate partners, such as waiting before reacting, not sharing every thought, driving around in the car to calm down, breathing deeply or asking for cool down time. Second, these immediate strategies of non-reactivity are accompanied by a triad movement of withdrawing, reflecting and rejoining with partners for dialogue. Particularly, the reflecting part appears to be a very deliberate and prolonged phase, as Laura (she/her, 28, pansexual) describes:

Laura: It's always important to me to first reflect on what happened. What was it actually? What it was that bothered me or what the conflict was and whether that's something that also has to do with him or more to do with me. And then to return into conversation with him.

Reflecting time for “sorting it out” is often specifically requested from a partner. It involves conscious emotional work thinking about how to resolve biographical issues, what a specific emotion might mean, what each partners’ role in conflict might be, and what to communicate in which words. The sorting process sometimes comes with specific strategies. Torben (he/him, 48, heterosexual) reports that he has an internal scale from 1 to 10 on which he rates how essential a conflict really is. Then he tries to get to the bottom of important topics on his own, before discussing them: “I try to capture that methodically somehow, with (..) writing it down, with sorting, with thinking about it, writing down lists for myself.”

When rejoining with a partner, some participants also report specific goals they work toward. These involve taking responsibility for own biographical vulnerabilities that became evident through reflection, or making an effort to step outside of the emotional “comfort zone” by showing vulnerability, “specially in one’s ugly, not very pretty moments” (Christine). Pia (they/them, 42, pansexual) reflects:

Pia: I think the more difficult thing is to be open with yourself and also talk about dark sides. That feels more difficult to me, and that is then…then I need to think on what is going on with myself, before I can communicate that.

Finally, different forms of self-restraint regarding emotional needs toward intimate partners are visible in our interviews. For one thing, participants reported accepting boundaries and turning to self-regulation when a partner needs time to process or cannot provide emotional support.

Erika: With [partner 2] I rather have the feeling […] that, when he is distant, [he] simply has a lot on his plate and therefore simply has no capacity for this. And then it is fully, absolutely okay for me, because then he needs time."

Self-restraint is also exerted with the intention to protect one partner’s boundaries, even if this means not receiving emotional support from this or the other partner. This can occur when a partner does not want to hear (or tell) things about the relationships the participant would actually love to share (or know). This can be by general agreement, or after individual assessment, like when Mirjam (she/her, 34, bisexual, heteroromantic) decides not to disclose certain things that her partner may “just be chewing on for the next week.” Self-restraint is also visible in Neles (they/them, 26, pansexual) case:

Nele: But there are also days when I come back from a date and say "Hey, I just had a really cool date" and the other person then says "Okay, I can't hear that right now, but I'd be open to your telling me about it tomorrow". It is in this sense that we actually try to always communicate our boundaries in the moment as much as we can, and, right, just to stay open and honest while communicating anyway, even if you maybe can't [deal with something] in that moment.

Sometimes, boundaries are created toward one partner to help another partner regulate or resolve conflict with this partner, as in Melanie’s (she/her, 25, queer) case:

Melanie: I think it's very important that when I'm in a conflict situation that's more or less resolved, that I don't, let's say, immediately run off to [partner 2] and say "ah, it's totally exhausting with [friend], I'm hanging out with [partner 2] in the [region] area for a week," but rather to say "hey, here's the thing: we had planned a week then and there, but I still need time here to sort things out and do things." And then to clarify that to the end and then, if necessary, even to have some additional shared time afterwards in this clarified state, which is then the basis for me being able to go away again.

### Self-Congruent Lifestyle

A third theme played upon by our participants was the need for a self-congruent lifestyle and its fulfillment. For one thing, many of our participants mentioned that their polyamorous relationship model helped them “be who I am,” “simply being oneself,” that they felt authentic in this lifestyle. Nele mentions that she doesn’t have to be ashamed or disguise herself in any way when she finds another person sexually attractive or develops feelings for this person. Katja (she/her, 37, bisexual) shares that she does not have to decide against her own feelings, because

Katja: […] you no longer have to leave someone for someone else, that there is no longer an either or […]. That you only decide against someone because you just don't have feelings for that person anymore, but not because you have feelings for other people as well.

Other aspects of “being oneself” include not having to wear a mask, being able to speak as openly as one wants to in the relationship (in contrast to the relationship model “don’t ask, don’t tell”), and having one’s unique constellation of needs met:

Kim: This sounds very selfish, but it simply is like this: [I like] that I feel good, that all my needs for passion and lightness are met. That I have the space to arrive somewhere with my whole “being so much/many.”

The urge to remain true to one’s own unique identity is so strong that it is pursued even when validation by partners is lacking. For instance, Mirjam (she/her, 34, bisexual/heteroromantic) describes how her partner would like to see her change:

Mirjam: And sometimes he says: “Hey, don't you want to somehow pay more attention to becoming calmer […]?” And that's when I always say “I'm just different from you, let me be the way I am.” Otherwise, that is something like… not exactly invasive…but I don't want to be patronized.

The autonomy to be oneself was also visible for other aspects of self-expression, such as resisting the pressure to wear makeup, lose weight, or to move in with someone. (Interestingly, one frequently stated motivation for polyamory, namely bisexuality, was not explicitly mentioned in our interviews.) In many cases, high differentiation is visible and is upheld in the face of tension, criticism for being too independent, and dealing with being misunderstood. In some cases, this independence does not appear as a struggle, but as a serene upholding of own autonomy which promotes a relaxed response in partners, too, as in Ahmet’s (he/him, 48, heterosexual) case:

Ahmet: Well, I've actually had situations where my girlfriend or my wife were jealous. And I think that because it's just not an issue for me, I can accept it quite well that they are just having a feeling. And I don't have to wipe it away, I don't have to stand up to it or anything like that. And that way it doesn't become such a big issue.

In other cases, self-congruence is upheld despite the threat of a breakup or intense negative affect from partners such as jealousy and envy. Jens (he/him, 24, bisexual) describes it like this: “In the end, I had to decide that this is the path I want to walk. That led to the end of that [past] relationship, which was my last monogamous relationship.”

Second, there is joy in expressing, exploring and developing one’s own facets of personality. Having several simultaneous relationships means experiencing the self in new ways through different relationship dynamics, thus enabling a broader self-expression. Michelle feels, that in her second relationship,

Michelle: …another part of me can be expressed that also wants to be seen and is important. The beauty of being with [partner 2] is that I can try to let that out […] and then I have enough strength to tackle other things. And yet, I do not have to hide […] or play a role.

Other participants mention interests or skills that one partner has to offer, while the other does not. For some, the best part of this aspect is discovering and exploring novelty: they benefit from exploring new persons physically, following what is interesting and exciting about another person, or simply relishing the complete “newness” of a person (as opposed to discovering “new sides” of a well-known persons’ personality). For Bettina (she/her, 35, pansexual), keeping the life plan itself open to change and “newness” is a key ingredient of self-congruence:

Bettina: Together with the people who are most important to you, you can reflect on this: How do I want to live? How do I want to shape our life? What can I bring into this partnership? What can another partner possibly also bring to an existing constellation? How do we feel, together and individually? There is this element of communication and the fact that it's never fixed or stuck anywhere, that's just awesome.

Thus, and finally, for our interviewees, the polyamorous lifestyle provides ample material for growing, learning about communication, and learning from different role models. This personal development process is described as hard work, but rewarding:

Zoe (she/her, heteroflexible): Of course, I also came into contact with many difficult patterns, and it takes a lot of work to somehow dissolve them or find a way. But at the same time, somehow, I find it is totally valuable to have done that, because yes, just because of this growth, that happens then.

Christine: I really believe that […] this closeness and possibility of being in contact with many different people on a really intimate level […] somehow makes life very rich [...]. And the other part is that, together with other people, you invest so much time and energy into this kind of communication, and by doing so get to know many different levels of feelings, […] have insight into other people’s lives and […]–the results in terms of human competence, they then also flow into other areas of life.

Next to the aspect of acquiring social and emotional skills, part of personal growth is coming to terms with “the fact that there can be several persons in my life to whom I feel attracted and whom I can love” (Ahmet).

### Valuing Freedom

The last common theme emerging in our interviews is “freedom.” Feeling free and liberated was frequently mentioned. Prison and cage metaphors abounded, leaving the impression that the quality of freedom in this theme is not so much a “freedom to”-quality (for instance, to live in an authentic manner), but an emphatic “freedom from”-quality. This was primarily experienced as freedom from societal scripts, norms and expectations regarding intimate relationships. Participants describe as liberation the fact that they are no longer restricted in the way they live out their love for others. They mention that they had this “diffuse feeling of being imprisoned and of dissatisfaction” (Pia) in monogamous relationships, while now, it is possible to let out “this insane abundance of love and feelings, which exists and which has permission to be here in all its facets” (Laura). However, it is not only about freedom from the norm of monogamy. Lisanne values the opportunity to live outside of the standard script of how relationships generally are supposed to be conducted:

Lisanne: [Getting] away from this rigid “this is how it must be,” and to have so many possibilities instead. […] There is so much between friendship and romance. And I can shape this in every relationship […]. And I don't need to have a certain pattern or standard scheme into which I must press everyone. Every person has different needs […] and by consensus with all I can shape my relationship and my life exactly as it suits me.

This includes open negotiation and clarity about boundaries and rules, which, according to Ahmet is opposed to the obscure and unspoken expectations in exclusive relationships, where he constantly wonders where he is crossing boundaries: “And I find that in a polyamorous relationship, because of the discussion and negotiation of boundaries, […] it is liberating for me […] to know what the boundaries are.”

Freedom from the overwhelming expectation of being “the one and only” for someone also came up repeatedly. One participant mentions that he does not want to be the “monopolist,” unifying everything for one partner. Another describes it as totally unfair to “dump all of that on one person.” Kim states: “I am very much and big and I need very much and I am very demanding–and I have had the experience that one person alone cannot fulfill that.” Nicole, in turn, does not want to feel the pressure of being “everything” for a person and trying to give this person anything they want. Particularly, with regard to sexual wishes she is happy to try things, but she wouldn’t feel comfortable in the (hypothetical) case that her partner.

Nicole: …would say “but I can't live without it”. Then, please, look for someone, […] who does that with you and who is up for it–I am not up for it. (...) Yes, [I like the fact] that several people can cover it.

Finally, there is freedom from structural and emotional fusion with a partner. Structural fusion refers to life situations, financial independence, and standing on one’s own two feet. Laura lives alone and does not want to link her finances with other people or weave a life structure together, such as her own apartment. Pia finds that this feeling of freedom is a prerequisite for her get deeply involved in relationships. She appreciates that “We can all be completely ourselves, our own life, so to speak, yes, lead our own life, although it is totally interwoven with that of others. And yet we can still have this, this intense closeness."

Freedom from emotional fusion refers to not being exclusively emotionally dependent from one partner, or vice versa. Feeling uncomfortable with this was frequently stated. Since emotional dependence is a position from where emotional demands and pressure could ensue, rejecting emotional fusion comes with the freedom to resist external control, demands and intrusion. Torben reports that he expects the liberty to be self-determined within the boundaries of negotiated agreements, whereas his wife “would like to direct him a bit, how to behave in different situations.” She exerts pressure (which he finds intrusive) by saying: "Now you've made this and that mistake and that's why I'm feeling bad. So stop it, please behave like this and like this, then I'll feel better again." That […] is what I would consider crossing the line.”

Our participants feel that no right exists to be held captive or hold other people in captivity. Melanie calls this the “toxic way mono-persons interpret their relationships and what people can do in these.” In contrast, the existence of the polyamorous lifestyle and of other partners makes participants feel less obligated to conform to relational pressure, as in the case of Erika (she/her, 24, bisexual):

Erika: I actually find it very interesting sometimes, the things he demands about my relationship with [partner 2]. To which I then sometimes say "Okay stop, somehow, why (laughs) do you demand […] that, when I just kind of accepted it in your other relationship?”

The polyamorous people at hand feel strongly about preserving boundaries, privacy and secrets, deciding over one’s own time and saying no to demands. One interviewee also does not like it when their partner imitates them or constantly uses “we”–instead of “I”-clauses. Generally, reflecting on pressure, obligation and fusion, many participants explicitly express how highly they value autonomy in general and in their partners: “I think we both attribute a certain autonomy to each other and also find that very important in the other” (Nele). “I value independence very much, when people can provide for and be with themselves, when they don't always need other people. And above all, always need me” (Laura). “I find independence rather a big advantage in a person.” (Melanie).

Torben: Autonomy is actually kind of a chill pill for me. So, when I notice that my wife or my girlfriend is acting completely autonomously and independently, then that's more pleasant for me than that I would see it as a problem.

However, in this context several participants comment on balancing autonomy with connection:

Christine: You are not incomplete without the other person nor you have such a […] strong dependency. But there is an interdependence, an interweaving with each other and I think [Partner 1] and I are interwoven very beautifully. […] Certain dependencies simply arise in a partnership, emotionally, when you live together. […] We can balance [them] very, very well and can still say that this emotional freedom that we have, gives us the possibility to be very, very close and want this closeness.

## Discussion

Different theoretical lenses provide different facets for conceptualizing autonomy in intimate relationships. (1) Viewed from self-determination theory, it can be understood as the capacity for self-governed agency in interactions with romantic partners. (2) Viewed from attachment theory, it is the emotional freedom to experience both togetherness and separateness with romantic partners from a position of inner security and trust. (3) Viewed from differentiation theory, it consists of living in accordance with one’s own unique identity even when this is challenged by expectations of romantic partners or wider societal norms. Persons in polyamorous constellations enjoy unusually high degrees of freedom to entertain sexual and romantic relationships compared to monogamous individuals. However, they also face the complex challenge of balancing this autonomy with needs for intimacy and closeness in the context of non-exclusivity.

**Fig. 1:**
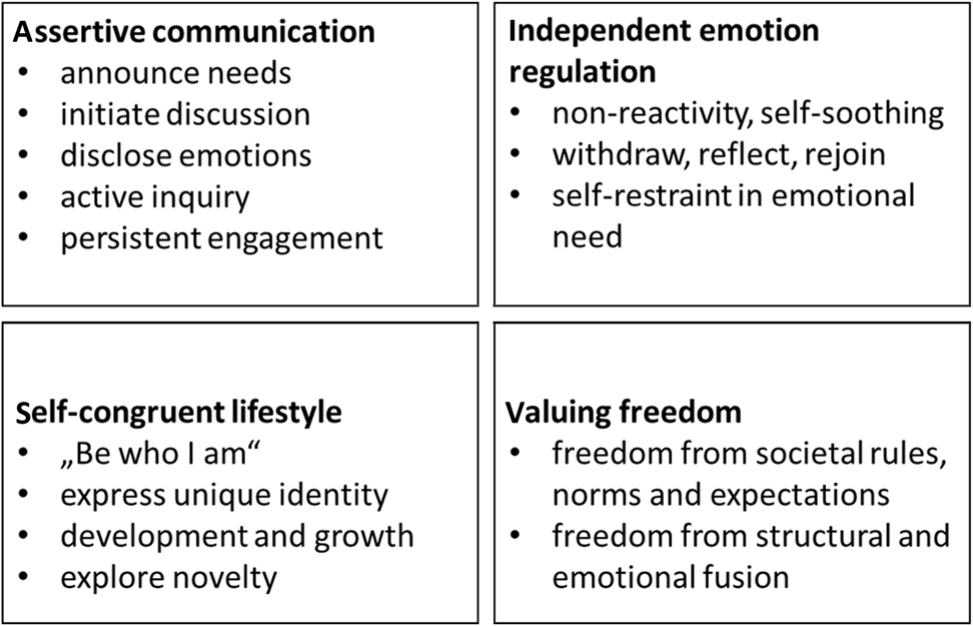
Facets of autonomy in polyamorous relationships

Using qualitative data, the present study explored if and how personal autonomy is created, expressed, and prioritized in polyamorous relationships. Four central aspects of autonomy in polyamory emerged: (1) assertive communication, (2) independent emotion regulation, (3) self-congruent lifestyle, and (4) valuing freedom. Figure 1 summarizes sub-facets of each aspect.

Due to the limitations of our study, these findings are tentative and provisional. We used qualitative data from a relatively small number of self-selected and non-representative participants. We sampled predominantly via the snowball system, with no precise knowledge of how study information was forwarded. This may have contributed to the homogeneity of our participants, which was on average young, female, queer, and convinced of polyamory as a way of life. We did not ask about ethnicity, disabilities, residential situation or other socioeconomic attributes, relationship duration or the specific organizational form of the multi-partner constellation (e.g., "polycule" or "quad"). Instead, a comparative approach was chosen that distinguished between a primary and secondary attachment partner, thereby also leaving many questions unanswered. Differences between hierarchically and non-hierarchically organized polyamorous networks should be given more focus in future studies (Flicker et al., 2021). Also, the multiple facets of subjectivities at the intersection of class, gender, race and other sociostructural attributes fall short in our analysis. Any kind of attachment manifests itself within a dynamic shaped by multiple divisions that were not captured in our study, thus limiting our findings. Our sample was predominantly female and queer, a context that will be referred to in the following discussion. Individuals with more ambivalent attitudes or more negative experiences, older people, other persons identifying as heterosexual or male may have a different view on the subject. Future quantitative studies must examine whether the findings of our study also apply to larger and broader populations and can thus be generalized. Nevertheless, the thoughtful, multifacetted and in-depth answers of our participants provide insights on “doing autonomy in polyamory” and therefore preliminary answers to the research questions raised above.

### In What Ways Is Personal Autonomy Created and Expressed in Polyamorous Relationships?

Autonomy is created and expressed through an assertive communication style. Navigating difficult discussions in an open, confident, and constructive manner while at the same time maintaining the connection during disagreement is a hallmark of autonomy (Loeb et al., 2020). It encourages conversation partners to also come forward, speak up and engage in open and assertive communication. Our interview material reflects high levels of willingness and ability to communicate in an autonomous way. Participants appeared to enjoy the challenge of announcing their needs and initiating prolonged discussions around them. It could be assumed that this, at least in part, may be owed to educational chances. In fact, criticism toward polyamory discourse often centers around the observation that CNM often occurs in educated, white individuals, thus naturalizing ways of relating that may solidify class privileges instead of improving reality for many identities (Sheff & Hammers, 2011).

In accordance with SDT, participants’ actions were described as self-initiated and self-directed as well as consistent with their own agenda. This also included the ability to actively inquire about their conversation partner’s viewpoint, thus creating space for exploring potentially conflicting interests of their partners. All of these behaviors are described as relevant to autonomy in interaction, for instance in Allen et al.’s (2000) widely used Autonomy and Relatedness Coding System. This manual also lists behaviors that prevent autonomy, such as withdrawing one's own point of view without having been convinced, attacking, or exerting coercive pressure. These kinds of behaviors were rarely mentioned in our interviews. Instead, participants appeared feeling comfortable with the intensity of their relationship conversations. They were not interested in prevailing “against all odds”; instead, they used strategies of disclosing emotions and hence, vulnerability in explaining their motivations and intentions, and persistent engagement to make sure, creative and consensual solutions could be found.

Autonomy is also created and expressed through self-directed, independent emotion regulation, which is an important skill in conducting non-violent communication. Emotion regulation refers to any process or action by which an individual influences their emotions or their emotional expression. An individual can regulate emotions in multiple ways, including seeking or avoiding specific situations, ways to think about personal experience and to express feelings. Some forms of regulation are associated with greater well-being, such as cognitive reappraisal, mindfulness, and acceptance (Bröning & Brandt, 2022; Vøllestad et al., 2012), whereas other strategies, such as suppression, are associated with poorer psychological outcomes (D’Agostino et al., 2017). Emotion regulation skills in the context of intimate relationships are connected to attachment security, where autonomy means being able to flexibly use self and other for purposes of soothing when dealing with a threatening stimulus evoking negative affect. In this material, several aspects of self-regulation emerged. Participants described strategies such as non-reactivity and self-soothing in a heated conflict, the triad strategy of withdrawing, reflecting and rejoining the discussion, and self-restraint with regard to their partners emotional boundaries. Throughout the interviews, negative affect, especially envy and jealousy, was perceived as a challenge that can be managed to a certain degree between partners (e.g., through clear rules and transparency), but must primarily be dealt with self-responsibly (e.g., by using self-regulatory strategies in moments of jealousy; Farrell, 2022).

These results point to the fact that emotion regulation skills may be at the heart of keeping up multi-partnered constellations. Strong emotions may come up when one partner is not available, or a partner may not be the right person to address in a specific situation. Also, polyamorous constellations have to deal with conflict still framed as irresolvable in current discourses—a partner having a sexual connection with someone else on a regular basis. Certainly, these are “societally unstandardized conflicts” that frequently lead to breakup in monogamous constellations. Experiencing or dealing with affect in unusual ways has even produced new feeling words such as “fribbly” or “compersion” (Farrell, 2022). Since more people are involved, vulnerability in poly constellations may increase, potentially resulting in more power struggles. However, as Gregoratto (2020) mentions, it could also foster the development of new coping strategies with regard to ambivalence or doubt, which may decrease possessiveness and egoism. In that sense, while critics rightly contend the existence of “autonomy per se” in a society with class differences, polyamory may in some cases represent a “social laboratory” (Gregoratto, 2020, p. 14), where relational autonomy can be practiced and attachment security can be increased over time. Deri (2015) describes the “feeling rules” used by queer poly women as the fruit of cultural experiments that form an important asset and function as a repository of response strategies for resolving potential conflicts around jealousy.

### Are Autonomy-Related Needs Fulfilled in Polyamorous Relationships?

Our participants argued that their relationship model enabled them to live a self-congruent lifestyle, where they can “be who I am,” express unique identity, explore novelty, and develop their personality. This resonates with the connection between autonomy, unique identity and the need for authenticity drawn from differentiation theory. Intimate relationships provide an arena for developing and expressing one’s identity—for instance by living a polyamorous lifestyle—and stay true to it even when it is challenged. This challenge may be especially pronounced in multi-partner constellations. Kauppi (2021) calls polyamory a “trial by fire for differentiation.” She describes the difficulty of a situation where an individual with simultaneous partners may lead a dual life with two households and cultures. This lifestyle challenges individuals to hold on to their “internal compass,” while staying “relationally connected with integrity with two or more different people, each of whom has their own priorities and beliefs” (p. 324). In this sense, polyamory can be viewed as an opportunity for growth and building relational strength for those who endorse this challenge and are equipped to deal with it. At the same time, it facilitates living specific aspects of personalities that may not be well-reflected in monogamous relationships such as novelty/sensation seeking, openness to experiences, the tendency to self-actualize in relationships, widespread interests, and the capacity to love several intimate partners. These traits are often stigmatized by traditional monogamous societal norms that discourage exploration outside the dyad. Being able to express them may place a liberating potential for authenticity at the heart of erotic autonomy, whether expressed within a monogamous, a “mono-flexible,” or a polyamorous relationship.

This may be especially valid for women as a group, because, historically, their sexual pleasure has been systematically oppressed (Vance, 1984), and for bi- and pansexual individuals, for whom in some cases, multi-partner relationships may serve as an act of agency and empowerment (Baumgartner, 2017; Klesse, 2005). Sexual, gender, and other minorities continue to face stigma, promiscuity discourses, sexual harassment, and erasure (Korinth et al., 2024) They have responded to these deep-rooted identity challenges through collective community building and activism, and may be bringing this strength to the field of CNM, considering the overlap between sexual, gender and romantic minority persons. Schechinger (2018) studied CNM and found that 10% of his sample identified as genderqueer, and only 26% of his sample as heterosexual. Valuing freedom from restrictions, questioning scripts and adopting new relational approaches may have prompted queer persons to pursue multi-partner relationships (Vaughan & Witherspoon, 2022), while having learned to hold on to an own identity in the face of obstacles may have equipped them with the competencies to maintain them, such as emotional strength, genuineness, and social intelligence.

In sum, polyamory may offer a wide range of autonomy options for individuals, but it requires extensive skills (or skill-building) in the area of relational competence, cognitive independence, dealing with uncertainty and ambiguity, self-regulation, resistance to societal norms and stigma, as well as the ability to hold multiple perspectives and “truths.” Allen (2007) attributes autonomy to the twin capacities of self-reflection and self-transformation. Almost certainly, constant reminders about the potential fragility and impermanence of intimate relationships are not a life setup many people might choose for themselves. From a societal perspective, intersecting class differences may play a role in limiting individuals’ choice of this relationship model, since not everybody has the same opportunity for developing cognitive, social, emotional skills, or holds the same relationship values. It should be mentioned again, though, that these skills are valuable in any post-modern relationship due to the high psychological expectations toward love in place. Thus, Klesse (2018) rightly recommends a multiscalar perspective on autonomy that encompasses “individual, intersubjective, multiple socio-political and socio-economic dimensions within and across the realms of gender, sexuality, race and class” (p.2).

### How Important Is Personal Autonomy for Polyamorous Individuals?

Autonomy proved to be is very meaningful to polyamorous individuals, who value freedom on a relational level, by avoiding structural and emotional fusion, and on a societal level, by feeling free from societal norms and expectations. Participants described feeling liberated from the confinement of having to live in a standard way, in an unauthentic way, or by suppressing the abundance of love and feelings they were experiencing. However, in accordance with attachment theory, the need to include, respect, and take into account existing connections when engaging with new persons was also frequently expressed. Farrell (2022) underlines the fact that polyamory is a way of meeting needs for autonomy without discounting or leaving existing partners, for instance in the case of sexual incompatibility. Participants valued freedom from expectations: not wanting to be the monopolist, i.e., “everything” for one partner, and that they did also not want to be dependent on one partner to fulfill all of their needs. Finally, our participants rejected the notion that a person can “own” another person, tell them what to do or how to be, or unilaterally impose rules on them. While the interviewees were willing to not only receive, but also provide this freedom, their statements reflected that this does not come easy, but involves an extensive amount of “relationship work,” self-regulation, communication, and reflection.

This is valuable information for persons considering embarking on the polyamorous journey. It provides counsellors and therapists with topical material and guidance as to potential areas of conflict and growth in romantic relationships. It also hints at who might be actually be better or less suited to successfully navigate a polyamorous lifestyle. It challenges mental health practitioners to move from a deficit-oriented perspective on CNM as infidelity and inherently unhealthy lifestyle to a differential perspective. This is not a model for everyone. The relationship maintenance practices of CNM, such as honesty, sexual health practices, and friendship among romantic partners (Mogilski et al., 2023), may not be easy to implement for persons who feel insecure in attachments, or prefer to end them avoid emotional dispute. However, polyamory provides unique relationship opportunities for persons who highly value autonomy, connection, and novelty and are willing to invest money, time, and emotional effort into obtaining it. “Double pain, double gain”—this quote from one of our interviewees summarizes the impression conveyed by the rich and complex material on polyamory provided by the participants of this qualitative study.

## Data Availability

The datasets and codes generated during and/or analyzed during the current study are available from the author on reasonable request.

## References

[CR1] Ainsworth, M. D. S., Blehar, M. C., Waters, E., & Wall, S. N. (2015). *Patterns of attachment: A psychological study of the strange situation*. Psychology Press.

[CR3] Allen, A. (2007). *The politics of our selves: Power, autonomy, and gender in contemporary critical theory*. Columbia University Press.

[CR2] Allen, J., Hauser, S., Bell, K., McElhaney, K., Tate, D., Insabella, G., & Schlatter, A. (2000). *The autonomy and relatedness coding system*. Unpublished manuscript, University of Virginia, Charlottesville.

[CR4] Allen, J. P., Hauser, S. T., Bell, K. L., & O’Connor, T. G. (1994). Longitudinal assessment of autonomy and relatedness in adolescent-family interactions as predictors of adolescent ego development and self-esteem. *Child Development,**65*(1), 179–194. 10.1111/j.1467-8624.1994.tb00743.x 8131646 10.1111/j.1467-8624.1994.tb00743.x

[CR5] Andersson, C. (2022). Drawing the line at infidelity: Negotiating relationship morality in a Swedish context of consensual non-monogamy. *Journal of Social and Personal Relationships,**39*(7), 1917–1933. 10.1177/02654075211070556

[CR6] Balzarini, R. N., Dharma, C., Kohut, T., Campbell, L., Lehmiller, J. J., Harman, J. J., & Holmes, B. M. (2019). Comparing relationship quality across different types of romantic partners in polyamorous and monogamous relationships. *Archives of Sexual Behavior,**48*, 1749–1767. 10.1007/s10508-019-1416-7 31069571 10.1007/s10508-019-1416-7

[CR7] Balzarini, R. N., Shumlich, E. J., Kohut, T., & Campbell, L. (2018). Dimming the “halo” around monogamy: Re-assessing stigma surrounding consensually non-monogamous romantic relationships as a function of personal relationship orientation. *Frontiers in Psychology,**9*, 894. 10.3389/fpsyg.2018.00894 30008682 10.3389/fpsyg.2018.00894 PMC6034202

[CR8] Barker, M. (2005). This is my partner, and this is my… partner’s partner: Constructing a polyamorous identity in a monogamous world. *Journal of Constructivist Psychology,**18*(1), 75–88. 10.1080/10720530590523107

[CR9] Baumgartner, R. (2017). “I think I’m not a relationship person”: Bisexual women’s accounts of (internalised) binegativity in non- monogamous relationship narratives. *Psychology of Sexualities Review,**8*(2), 25–40.

[CR10] Baxter, L. A. (2006). Communication as dialogue. In G. J. Shepherd, J. St. John, & T. Striphas (Eds.), C*ommunication as…: Perspectives on theory* (pp. 101–109). SAGE Publications.

[CR11] Birnbaum, G. E., & Reis, H. T. (2019). Evolved to be connected: The dynamics of attachment and sex over the course of romantic relationships. *Current Opinion in Psychology,**25*, 11–15. 10.1016/j.copsyc.2018.02.005 29486255 10.1016/j.copsyc.2018.02.005

[CR12] Bowlby, J. (1979). The Bowlby-Ainsworth attachment theory. *Behavioral and Brain Sciences,**2*(4), 637–638.

[CR13] Braun, V., & Clarke, V. (2006). Thematic analysis revised—Final. *Qualitative Research in Psychology,**3*(2), 77–101. 10.1191/1478088706qp063oa

[CR14] Bricker, M. E., & Horne, S. G. (2007). Gay men in long-term relationships: The impact of monogamy and non-monogamy on relational health. *Journal of Couple & Relationship Therapy,**6*(4), 27–47. 10.1300/J398v06n04_02

[CR16] Bröning, S., & Brandt, M. (2022). “Mindful parenting” – Achtsamkeit in der Eltern-Kind-Beziehung. *Zeitschrift für Kinder-und Jugendpsychiatrie und Psychotherapie, 50*. 10.1024/1422-4917/a000853 35187975 10.1024/1422-4917/a000853

[CR15] Bröning, S. Clüver, A. & Gebhard, K. P. (2024). Facets of intimacy in consensually non-monogamous relationships. A qualitative interview study. *Zeitschrift für Sexualforschung, 37*(3), 133–141.

[CR17] Bröning, S., & Wartberg, L. (2022). Attached to your smartphone? A dyadic perspective on perceived partner phubbing and attachment in long-term couple relationships. *Computers in Human Behavior,**126*, Article 106996. 10.1016/j.chb.2021.106996

[CR18] Conley, T. D., Moors, A. C., Matsick, J. L., & Ziegler, A. (2013a). The fewer the merrier?: Assessing stigma surrounding consensually non-monogamous romantic relationships. *Analyses of Social Issues and Public Policy,**13*(1), 1–30. 10.1111/j.1530-2415.2012.01286.x

[CR19] Conley, T. D., Ziegler, A., Moors, A. C., Matsick, J. L., & Valentine, B. (2013b). A critical examination of popular assumptions about the benefits and outcomes of monogamous relationships. *Personality and Social Psychology Review,**17*(2), 124–141. 10.1177/1088868312467087 23175520 10.1177/1088868312467087

[CR20] Cubells-Serra, J., Sánchez-Sicilia, A., Astudillo-Mendoza, P., Escandón-Nagel, N., & Baeza-Rivera, M. J. (2021). Assumption of the myths of romantic love: Its relationship with sex, type of sex-affective relationship, and sexual orientation. *Frontiers in Sociology,**6*, Article 621646. 10.3389/fsoc.2021.621646 34095286 10.3389/fsoc.2021.621646 PMC8175080

[CR21] D’Agostino, A., Covanti, S., Rossi Monti, M., & Starcevic, V. (2017). Reconsidering emotion dysregulation. *Psychiatric Quarterly,**88*, 807–825. 10.1007/s11126-017-9499-6 28194549 10.1007/s11126-017-9499-6

[CR22] Deri, J. (2015). *Love’s refraction: Jealousy and compersion in queer polyamorous relationships*. University of Toronto Press.

[CR23] DeWall, C. N., Lambert, N. M., Slotter, E. B., Pond, R. S., Jr., Deckman, T., Finkel, E. J., Luchies, L. B., & Fincham, F. D. (2011). So far away from one’s partner, yet so close to romantic alternatives: Avoidant attachment, interest in alternatives, and infidelity. *Journal of Personality and Social Psychology,**101*(6), 1302–1316. 10.1037/a0025497 21967006 10.1037/a0025497

[CR24] Elitepartner. (2023). *ElitePartner Studie 2023*. https://www.elitepartner.de/studien/.

[CR25] Fairbrother, N., Hart, T. A., & Fairbrother, M. (2019). Open relationship prevalence, characteristics, and correlates in a nationally representative sample of Canadian adults. *Journal of Sex Research,**56*(6), 695–704. 10.1080/00224499.2019.1580667 30932711 10.1080/00224499.2019.1580667

[CR26] Farrell, R. M. (2022). Polyam affect: Working with emotions in CNM. In M. D. Vaughan & T. R. Burnes (Eds.), *Handbook of consensual non-monogamy: Affirming mental health practice* (pp. 74–96). Rowman & Littlefield.

[CR27] Feldman, R. (2017). The neurobiology of human attachments. *Trends in Cognitive Sciences,**21*(2), 80–99. 10.1016/j.tics.2016.11.007 28041836 10.1016/j.tics.2016.11.007

[CR28] Fern, J. (2020). *Polysecure: Attachment, trauma and consensual nonmonogamy*. Thorntree Press LLC.

[CR29] Fisher, H. (2000). Lust, attraction, attachment: Biology and evolution of the three primary emotion systems for mating, reproduction, and parenting. *Journal of Sex Education and Therapy,**25*(1), 96–104. 10.1080/01614576.2000.11074334

[CR30] Flentje, A., Heck, N. C., Brennan, J. M., & Meyer, I. H. (2020). The relationship between minority stress and biological outcomes: A systematic review. *Journal of Behavioral Medicine,**43*, 673–694. 10.1007/s10865-019-00120-6 31863268 10.1007/s10865-019-00120-6 PMC7430236

[CR31] Flicker, S. M., Sancier-Barbosa, F., Moors, A. C., & Browne, L. (2021). A closer look at relationship structures: Relationship satisfaction and attachment among people who practice hierarchical and non-hierarchical polyamory. *Archives of Sexual Behavior,**50*(4), 1401–1417. 10.1007/s10508-020-01875-9 33956295 10.1007/s10508-020-01875-9

[CR32] Friedman, M. (1998). Romantic love and personal autonomy. *Midwest Studies in Philosophy,**22*, 162–181. 10.1111/j.1475-4975.1998.tb00336.x

[CR33] Gagliardi, M. (2021). How our caregivers shape who we are: The seven dimensions of attachment at the core of personality. *Frontiers in Psychology,**12*, Article 657628. 10.3389/fpsyg.2021.657628 34276482 10.3389/fpsyg.2021.657628 PMC8280313

[CR34] Garner, C., Person, M., Goddard, C., Patridge, A., & Bixby, T. (2019). Satisfaction in consensual nonmonogamy. *The Family Journal,**27*(2), 115–121. 10.1177/1066480719833411

[CR35] Givertz, M., Woszidlo, A., Segrin, C., & Knutson, K. (2013). Direct and indirect effects of attachment orientation on relationship quality and loneliness in married couples. *Journal of Social and Personal Relationships,**30*(8), 1096–1120. 10.1177/0265407513482445

[CR36] Gregoratto, F. (2020). The passionate nature of freedom: From Hegel to Dewey and Adorno; From this to another country. In P. Giladi (Ed.), *Hegel and the Frankfurt School* (pp. 242–264). Routledge.

[CR37] Gregoratto, F. (2021). Elf Thesen zur Polyamorie [Eleven theses on polyamory]. *Philosophie Magazin Nr.,**04*, 38–41.

[CR38] Hacking, I. (1999). *The social construction of what?* Harvard University Press.

[CR39] Hardy, N. R., & Fisher, A. R. (2018). Attachment versus differentiation: The contemporary couple therapy debate. *Family Process,**57*(2), 557–571. 10.1111/famp.12343 29363747 10.1111/famp.12343

[CR40] Haupert, M. L., Gesselman, A. N., Moors, A. C., Fisher, H. E., & Garcia, J. R. (2017). Prevalence of experiences with consensual nonmonogamous relationships: Findings from two national samples of single Americans. *Journal of Sex & Marital Therapy,**43*(5), 424–440. 10.1080/0092623X.2016.1178675 27096488 10.1080/0092623X.2016.1178675

[CR41] Hayes, S. C., Strosahl, K. D., & Wilson, K. G. (1999). *Acceptance and commitment therapy*. Guilford Press.

[CR42] Hazan, C., & Shaver, P. (1987). Romantic love conceptualized as an attachment process. *Journal of Personality and Social Psychology,**52*(3), 511–524. 10.1037/0022-3514.52.3.511 3572722 10.1037//0022-3514.52.3.511

[CR43] Hnatkovičová, D., & Bianchi, G. (2022). Model of motivations for engaging in polyamorous relationships. *Sexologies,**31*(3), 184–194. 10.1016/j.sexol.2022.03.003

[CR44] Katz, M., & Katz, E. (2022). Reconceptualizing attachment theory through the lens of polyamory. *Sexuality & Culture,**26*(2), 792–809. 10.1007/s12119-021-09902-0

[CR45] Kauppi, M. (2021). *Polyamory: A clinical toolkit for therapists (and their clients)*. Rowman & Littlefield.

[CR46] Kernis, M. H., & Goldman, B. M. (2006). A multicomponent conceptualization of authenticity: Theory and research. *Advances in Experimental Social Psychology,**38*, 283–357. 10.1016/S0065-2601(06)38006-9

[CR47] Kerr, M. E., & Bowen, M. (1988). *Family evaluation*. WW Norton & Company.

[CR48] Klesse, C. (2005). Bisexual women, non-monogamy and differentialist anti-promiscuity discourses. *Sexualities,**8*, 445–464. 10.1177/1363460705056620

[CR49] Klesse, C. (2007). Polyamory—von dem Versprechen, viele zu lieben. *Zeitschrift für Sexualforschung,**20*(04), 316–330. 10.1055/s-2007-981350

[CR50] Klesse, C. (2018). Toward a genealogy of a discourse on women’s erotic autonomy: Feminist and queer-feminist critiques of monogamy. *Signs: Journal of Women in Culture and Society,**44*(1), 205–231. 10.1086/698283

[CR51] Korinth, R., Bröning, S., & Martyniuk, U. (2024). Making the invisible visible: Experiences of identity (In)visibility in bi+sexual individuals in Germany. *Journal of Bisexuality, 24*, 508–533. 10.1080/15299716.2024.2364663

[CR52] Lampis, J. (2016). Does partners’ differentiation of self predict dyadic adjustment? *Journal of Family Therapy,**38*(3), 303–318. 10.1111/1467-6427.12073

[CR53] Levine, E. C., Herbenick, D., Martinez, O., Fu, T.-C., & Dodge, B. (2018). Open relationships, nonconsensual nonmonogamy, and monogamy among U.S. adults: Findings from the 2012 National Survey of Sexual Health and Behavior. *Archives of Sexual Behavior,**47*, 1439–1450. 10.1007/s10508-018-1178-7 29696552 10.1007/s10508-018-1178-7 PMC5958351

[CR54] Loeb, E. L., Davis, A. A., Costello, M. A., & Allen, J. P. (2020). Autonomy and relatedness in early adolescent friendships as predictors of short-and long-term academic success. *Social Development,**29*(3), 818–836. 10.1111/sode.12424 33692608 10.1111/sode.12424 PMC7938762

[CR55] Luyten, P., Campbell, C., Allison, E., & Fonagy, P. (2020). The mentalizing approach to psychopathology: State of the art and future directions. *Annual Review of Clinical Psychology,**16*, 297–325. 10.1146/annurev-clinpsy-071919-015355 32023093 10.1146/annurev-clinpsy-071919-015355

[CR56] Main, M., Hesse, E., & Goldwyn, R. (2008). Studying differences in language usage in recounting attachment history: An introduction to the AAI. In H. Steele & M. Steele (Eds.), *Clinical applications of the Adult Attachment Interview* (pp. 31–68). The Guilford Press.

[CR57] McIsaac, C., Connolly, J., McKenney, K. S., Pepler, D., & Craig, W. (2008). Conflict negotiation and autonomy processes in adolescent romantic relationships: An observational study of interdependency in boyfriend and girlfriend effects. *Journal of Adolescence,**31*(6), 691–707. 10.1016/j.adolescence.2008.08.005 18951625 10.1016/j.adolescence.2008.08.005

[CR58] Meyer, I. H. (1995). Minority stress and mental health in gay men. *Journal of Health and Social Behavior, 36*, 38–56. 10.2307/2137286 7738327

[CR59] Meyer, I. H. (2003). Prejudice, social stress, and mental health in lesbian, gay, and bisexual populations: Conceptual issues and research evidence. *Psychological Bulletin,**129*(5), 674–697. 10.1037/0033-2909.129.5.674 12956539 10.1037/0033-2909.129.5.674 PMC2072932

[CR60] Mikulincer, M., & Shaver, P. R. (2012). Adult attachment orientations and relationship processes. *Journal of Family Theory & Review,**4*(4), 259–274. 10.1111/j.1756-2589.2012.00142.x

[CR61] Mitchell, M. E., Bartholomew, K., & Cobb, R. J. (2013). Need fulfillment in polyamorous relationships. *Journal of Sex Research,**51*(3), 329–339. 10.1080/00224499.2012.742998 23541166 10.1080/00224499.2012.742998

[CR62] Mogilski, J. K., Rodrigues, D. L., Lehmiller, J. J., & Balzarini, R. N. (2023). Maintaining multi-partner relationships: Evolution, sexual ethics, and consensual non-monogamy. In J. K. Mogilski & T. K. Shackelford (Eds.), *The Oxford handbook of evolutionary psychology and romantic relationships* (pp. 461–486). 10.1093/oxfordhb/9780197524718.013.17

[CR63] Moors, A. C., Conley, T. D., Edelstein, R. S., & Chopik, W. J. (2015). Attached to monogamy? Avoidance predicts willingness to engage (but not actual engagement) in consensual non-monogamy. *Journal of Social and Personal Relationships,**32*(2), 222–240. 10.1177/0265407514529065

[CR64] Moors, A. C., Selterman, D. F., & Conley, T. D. (2017). Personality correlates of desire to engage in consensual non-monogamy among lesbian, gay, and bisexual individuals. *Journal of Bisexuality,**17*(4), 418–434. 10.1080/15299716.2017.1367982

[CR65] Morrison, T. G., Beaulieu, D., Brockman, M., & Beaglaoich, C. Ó. (2013). A comparison of polyamorous and monoamorous persons: Are there differences in indices of relationship well-being and sociosexuality? *Psychology & Sexuality,**4*(1), 75–91. 10.1080/19419899.2011.631571

[CR66] Neff, K. D., & Harter, S. (2003). Relationship styles of self-focused autonomy, other-focused connectedness, and mutuality across multiple relationship contexts. *Journal of Social and Personal Relationships,**20*(1), 81–99. 10.1177/02654075030201004

[CR67] Pieper, M., & Bauer, R. (2014). Polyamorie: Mono-Normativität–Dissidente Mikropolitik–Begehren als transformative Kraft? [Polyamory: Mono-normativity—Dissident micro politics—Desire as transformative force?]. *Journal für Psychologie*, *22*(1), 1–35.

[CR68] Plöderl, M., & Tremblay, P. (2015). Mental health of sexual minorities. A systematic review. *International Review of Psychiatry,**27*(5), 367–385. 10.3109/09540261.2015.1083949 26552495 10.3109/09540261.2015.1083949

[CR69] Reckwitz, A. (2019). Die Gesellschaft der Singularitäten [The society of singularities]. *Journal Für Politische Bildung,**9*(1), 10–17.

[CR70] Reynish, T., Hoang, H., Bridgman, H., & Nic Giolla Easpaig, B. (2023). Barriers and enablers to mental health help seeking of sexual, gender, and erotic minorities: A systematic literature review. *Journal of Gay & Lesbian Mental Health,**27*(2), 129–150. 10.1080/19359705.2022.2036666

[CR71] Ryan, R. M., & Deci, E. L. (2000). Self-determination theory and the facilitation of intrinsic motivation, social development, and well-being. *American Psychologist,**55*(1), 68–78. 10.1037/0003-066X.55.1.68 11392867 10.1037//0003-066x.55.1.68

[CR72] Ryan, R. M., Kuhl, J., & Deci, E. L. (1997). Nature and autonomy: An organizational view of social and neurobiological aspects of self-regulation in behavior and development. *Development and Psychopathology,**9*(4), 701–728. 10.1017/s0954579497001405 9449002 10.1017/s0954579497001405

[CR73] Ryan, W. S., & Ryan, R. M. (2019). Toward a social psychology of authenticity: Exploring within-person variation in autonomy, congruence, and genuineness using self-determination theory. *Review of General Psychology,**23*(1), 99–112. 10.1037/gpr0000162

[CR74] Schechinger, H. A., Sakaluk, J. K., & Moors, A. C. (2018). Harmful and helpful therapy practices with consensually non-monogamous clients: Toward an inclusive framework. *Journal of Consulting and Clinical Psychology,**86*(11), 879–891. 10.1037/ccp0000349 30335421 10.1037/ccp0000349

[CR75] Schippers, M. (2016). *Beyond monogamy: Polyamory and the future of polyqueer sexualities*. New York University Press.

[CR76] Schnarch, D. M. (2009). *Intimacy & desire: Awaken the passion in your relationship*. Scribe Publications.

[CR77] Sheff, E. (2020). Polyamory is deviant–but not for the reasons you may think. *Deviant Behavior,**41*(7), 882–892. 10.1080/01639625.2020.1737353

[CR78] Sheff, E., & Hammers, C. (2011). The privilege of perversities: Race, class and education among polyamorists and kinksters. *Psychology & Sexuality,**2*(3), 198–223. 10.1080/19419899.2010.537674

[CR79] Stefanou, C., & McCabe, M. P. (2012). Adult attachment and sexual functioning: A review of past research. *Journal of Sexual Medicine,**9*(10), 2499–2507. 10.1111/j.1743-6109.2012.02843.x 22759319 10.1111/j.1743-6109.2012.02843.x

[CR80] Taradash, A., Connolly, J., Pepler, D., Craig, W., & Costa, M. (2001). The interpersonal context of romantic autonomy in adolescence. *Journal of Adolescence,**24*(3), 365–377. 10.1006/jado.2001.0404 11476612 10.1006/jado.2001.0404

[CR81] Thompson, A. E., Bagley, A. J., & Moore, E. A. (2018). Young men and women’s implicit attitudes towards consensually nonmonogamous relationships. *Psychology & Sexuality,**9*(2), 117–131. 10.1080/19419899.2018.1435560

[CR82] Treboux, D., Crowell, J. A., & Waters, E. (2004). When “new” meets “old”: Configurations of adult attachment representations and their implications for marital functioning. *Developmental Psychology,**40*(2), 295–314. 10.1037/0012-1649.40.2.295 14979768 10.1037/0012-1649.40.2.295

[CR83] Vance, C. S. (1984). Pleasure and danger: Toward a politics of sexuality. In C. S. Vance (Ed.), *Pleasure and danger: Exploring female sexuality* (pp. 1–27). Routledge & Kegan Paul.

[CR85] Vaughan, M. D., & Burnes, T. R. (2022). *The handbook of consensual non-monogamy: Affirming mental health practice*. Rowman & Littlefield.

[CR84] Vaughan, M. D., & Witherspoon, R. G. (2022). Stronger together: CNM resilience, strengths, and growth. In M. D. Vaughan & T. R. Burnes (Eds.), *Handbook of consensual non-monogamy: Affirming mental health practice* (pp. 97–118). Rowman & Littlefield.

[CR86] Veltman, A., & Piper, M. (Eds.). (2014). *Autonomy, oppression, and gender*. Oxford University Press. 10.1093/acprof:oso/9780199969104.001.0001

[CR87] Vøllestad, J., Nielsen, M. B., & Nielsen, G. H. (2012). Mindfulness-and acceptance-based interventions for anxiety disorders: A systematic review and meta-analysis. *British Journal of Clinical Psychology,**51*(3), 239–260. 10.1111/j.2044-8260.2011.02024.x 22803933 10.1111/j.2044-8260.2011.02024.x

[CR88] Wetzel, D. (2014). *Polyamouröse Beziehungen als gelingende Lebensform? Resonanz-und anerkennungsanalytische Reflexionen*. [Polyamorous relationships as functioning way of life? Resonance and acceptance-analytical reflections]. DFG-Kollegforschergruppe Postwachstumsgesellschaften.

[CR89] Wosick-Correa, K. (2010). Agreements, rules and agentic fidelity in polyamorous relationships. *Psychology & Sexuality,**1*(1), 44–61. 10.1080/19419891003634471

